# High Resolution Topography of Age-Related Changes in Non-Rapid Eye Movement Sleep Electroencephalography

**DOI:** 10.1371/journal.pone.0149770

**Published:** 2016-02-22

**Authors:** Kate E. Sprecher, Brady A. Riedner, Richard F. Smith, Giulio Tononi, Richard J. Davidson, Ruth M. Benca

**Affiliations:** 1 Department of Psychiatry, University of Wisconsin, Madison, Wisconsin, United States of America; 2 Wisconsin Center for Sleep Medicine and Research, University of Wisconsin, Madison, Wisconsin, United States of America; 3 Neuroscience Training Program, University of Wisconsin, Madison, Wisconsin, United States of America; 4 Department of Psychology, University of Wisconsin, Madison, Wisconsin, United States of America; 5 Center for Investigating Healthy Minds, University of Wisconsin, Madison, Wisconsin, United States of America; Hôpital du Sacré-Coeur de Montréal, CANADA

## Abstract

Sleeping brain activity reflects brain anatomy and physiology. The aim of this study was to use high density (256 channel) electroencephalography (EEG) during sleep to characterize topographic changes in sleep EEG power across normal aging, with high spatial resolution. Sleep was evaluated in 92 healthy adults aged 18–65 years old using full polysomnography and high density EEG. After artifact removal, spectral power density was calculated for standard frequency bands for all channels, averaged across the NREM periods of the first 3 sleep cycles. To quantify topographic changes with age, maps were generated of the Pearson’s coefficient of the correlation between power and age at each electrode. Significant correlations were determined by statistical non-parametric mapping. Absolute slow wave power declined significantly with increasing age across the entire scalp, whereas declines in theta and sigma power were significant only in frontal regions. Power in fast spindle frequencies declined significantly with increasing age frontally, whereas absolute power of slow spindle frequencies showed no significant change with age. When EEG power was normalized across the scalp, a left centro-parietal region showed significantly less age-related decline in power than the rest of the scalp. This partial preservation was particularly significant in the slow wave and sigma bands. The effect of age on sleep EEG varies substantially by region and frequency band. This non-uniformity should inform the design of future investigations of aging and sleep. This study provides normative data on the effect of age on sleep EEG topography, and provides a basis from which to explore the mechanisms of normal aging as well as neurodegenerative disorders for which age is a risk factor.

## Introduction

Characterizing brain change across the healthy lifespan is important for understanding the mechanisms of normal aging as well as neurodegenerative disorders for which age is a risk factor. Because changes in sleeping brain activity reflect underlying changes in anatomy and neurophysiology, electroencephalography (EEG) during sleep can be a powerful tool for studying the aging brain. Sleep EEG is impacted by structural characteristics such as the number and health of cells and axons through which signals propagate. For example, power in the delta frequencies (1–4.5 Hz) correlates with the maturation of grey and white matter in adolescents as well as degeneration in elderly adults [1–3]. Sleep EEG also reflects functional properties; for example, power in the slow wave band (1–4.5 Hz) is modulated by synaptic strength [4] and synchronous firing [5], key mechanisms of information flow through neural networks [6]. EEG measures activity arising from neural ensembles on a millisecond timescale, capturing dynamic oscillatory activity that reflects functional connectivity. This fine temporal resolution is a key advantage of EEG over complementary imaging modalities such as magnetic resonance imaging (MRI) and positron emission tomography (PET) that are limited to time units of seconds or minutes. EEG is a particularly useful tool for imaging the brain in older populations, because it can be performed at the bedside and in individuals with contra-indications for MRI, such as metallic implants and claustrophobia. Furthermore, sleep EEG shows high test-retest stability within an individual across short time spans (days-weeks) [7–11] and recording during sleep allows the measurement of spontaneous brain activity, free from factors that complicate waking recordings across age ranges, such as movement, variations in attention and the ability to follow instructions. Thus sleep EEG can be used to examine structural and functional changes in dynamic neural networks across the life span.

Age-related alterations in grey and white matter structure, brain metabolism and connectivity show substantial regional variation [12–16]. Furthermore, it is becoming increasingly apparent that sleep does not occur uniformly throughout the brain. Rather, it can be a regional phenomenon, such that slow waves and spindles may occur in one brain region independent of activity in other regions [17], with a topography influenced by prior waking use [18,19]. Therefore, we hypothesized that the effect of age on sleep EEG would vary across the scalp. Across childhood, slow wave power shifts from posterior to frontal dominance, and correlates with anatomical cortical maturation [1,20]. In adulthood, previous work has suggested that age-related declines in EEG power during sleep are greatest in frontal derivations [21–25]. However, these studies had low spatial resolution (using only 4–20 electrodes), which could have missed or distorted the pattern of age effects on EEG. Regional variations in activity can be detected with greater accuracy when more electrodes are used; this has been empirically demonstrated using simulated and measured data [26,27]. Therefore we examined EEG topography with high spatial resolution by recording from 256 electrodes (high density EEG; hdEEG).

Prior studies assessing the effects of aging on sleep EEG topography included age as a binary variable, comparing young adults to older adults, and in the majority of studies the size of each group was small, ranging from 8 to 18. Although some studies have used regression to examine age-related changes in NREM spectral power [28], no studies have examined changes in topography throughout the life span. Aging is a progressive, rather than step-wise process, making the treatment of age as a continuous variable a more appropriate method of evaluating the brain changes associated with aging. Furthermore, midlife was largely neglected, with only one study including adults aged between 40 and 57 years,[21] and none including subjects between 30 and 40 years. It is important to investigate midlife as several studies indicate that it is a critical period; brain health at this age may predict resilience or vulnerability to disease in later life. For example, middle aged adults with obstructive sleep apnea (OSA) show greater cognitive deficits than younger adults with equally severe OSA [29,30] and mid-life vascular health predicts later development of dementia [31,32]. Understanding sleeping brain function throughout normal aging will provide an essential base from which to explore pathological patterns of brain aging.

The aim of this study was to characterize topographic changes in sleep EEG power across normal aging. We drew upon a pool of healthy adults aged 18–65 years old who had previously completed baseline studies in our laboratory using high density EEG.

## Methods

### Participants and Study Design

This study analyzed data collected from 92 adults aged 18–65 years (59 women) who had participated as healthy controls in protocols at the University of Wisconsin-Madison sleep laboratory (S1 and S2 Figs). Individuals were included in the analysis if they had undergone polysomnography (PSG) with hdEEG, without behavioral or pharmacological interventions. Participants were included only if they were free from neurological, major medical and sleep disorders including sleep disordered breathing (Apnea Hypopnea Index < 5/hr) and sleep related movement disorders (Periodic Limb Movements < 8/hr). Participants were excluded if they were taking drugs known to affect sleep (assessed by self-report). 54 participants (34 women) were drawn from a study of sleep and meditation, for which individuals aged 25–65 years were recruited through newspaper advertisements, email and distribution of recruitment flyers to meditation and wellness centers.[33] Only participants with no meditation experience were included in this analysis. 38 participants (22 women) were drawn from a study of normal sleeping brain activity[34–36], for which healthy adults were recruited through newspaper advertisements and word of mouth. Some participants were allowed to sleep until they awoke naturally while others were awakened at 6:30am. Therefore, to ensure that functionally similar sleep periods were examined across all individuals, participants were only included if they had experienced at least 3 sleep cycles during their PSG, with < 30% wake from sleep onset to the end of the third sleep cycle. For all participants, only the first 3 sleep cycles were analyzed. All participants were right-handed, defined by the Edinburgh Handedness Scale.[37]

Each participant’s medical and psychiatric histories were collected through an initial phone screening, followed by a thorough in-person screening involving questionnaires assessing general medical history, anxiety and depression, and a modified version of the Sleep Disorders Questionnaire[38] with 16 questions assessing symptoms of sleep apnea and periodic limb movement disorder. All participants were instructed to maintain regular sleep-wake schedules during the week prior to the study, which was confirmed with sleep diaries and wrist-worn actigraphy (Actiwatch, Mini-Mitter, Bend, OR), and to refrain from consuming caffeine and alcohol on the day of the study. Participants arrived at the laboratory between 7 and 9 pm, were set up with sensors for the sleep study (approximately 45 minutes), then went to bed within one hour of their usual bed time. They were allowed to sleep undisturbed until 6:30 am or until they awoke naturally. All data were drawn from baseline study visits; no pharmacological or behavioral interventions were performed. All participants provided informed consent, and protocols were approved by the Institutional Review Board of the University of Wisconsin-Madison.

### Polysomnography

Sleep was evaluated in all participants with standard PSG monitoring including electrooculogram (EOG), sub-mental electromyogram (EMG), electrocardiogram (ECG), bilateral tibial EMG, respiratory inductance plethysmography, pulse oximetry and a position sensor using a customized Alice 5 System (Philips Respironics, Murrysville, PA). Simultaneously, high density electroencephalography (hdEEG) was recorded from 256 channels with vertex referencing using NetStation software (Electrical Geodesics Inc., Eugene, OR). Sleep was scored by a registered sleep technologist according to AASM scoring guidelines [39] using Alice^®^ Sleepware (Philips Respironics, Murrysville, PA) and then reviewed by a Sleep Medicine physician certified by the American Board of Medical Specialties (RMB). Sleep staging was performed using 6 hdEEG channels located at approximate 10–20 locations (F3, F4, C3, C4, O1, O2), re-referenced to the mastoids.

### HdEEG Recordings

HdEEG signals were sampled at 500 Hz and referenced to the vertex, using a NetAmps 300 amplifier and NetStation software (Electrical Geodesics Inc., Eugene, OR). In NetStation a first-order high-pass filter (0.1 Hz) was applied to mimic common hardware analog filters and to eliminate low frequency drift. Data were filtered in MATLAB (The MathWorks Inc., Natick, MA) using first order high pass (0.1 Hz) to remove low frequency artifact associated with sweating, followed by a band-pass filter (Kaiser type, 1–50 Hz). The data were then average-referenced to the mean voltage across all good channels.

Analyses were performed on the first 3 sleep cycles only, to maximize equivalence between participants. Spectral analysis of NREM sleep was performed for each channel in six-second epochs (Welch’s averaged modified periodogram with a Hamming window). A semi-automatic artifact rejection was conducted to remove six-second sleep epochs that exceeded thresholds for individual channels based on the mean power in either low (1–4 Hz) or high (20–30 Hz) frequency bands. EEG channels in which artifacts affected most of the recording were subsequently removed. Average spectral density across NREM sleep was computed in six standard frequency ranges from the literature and our own studies:[40–42] delta (1–4.5 Hz), theta (4.5–8 Hz), alpha (8–12 Hz), sigma (12–15 Hz), beta (15–25 Hz) and low gamma (25–40 Hz). The frequency cutoffs used to distinguish between slow and fast spindles vary throughout the literature [23,40,43]. Therefore to explore differences between fast and slow spindle frequencies without selecting arbitrary cutoffs, spectral density was computed in 1 Hz bins from 9 to 16 Hz.

### Statistical Analysis

Preliminary analyses were performed to ensure no violation of the assumptions of normality, linearity and homoscedasticity. The relationship between age and sleep architecture across the first 3 sleep cycles was assessed using Pearson product-moment correlation coefficient. All-night sleep architecture is not reported, because the protocols were not designed to assess this. For comparison of spectral data and for initial topographic visualization, subjects were divided into 5 age groups: 18–24, 25–34, 35–44, 45–54 and 55–64 years. Each polysomnographic variable was analyzed separately in a two-way ANOVA with factors age group and gender. Significant main effects were assessed with post-hoc Tukey tests, with p<.05 considered significant. Spectral power density in 1/6 Hz width bins was compared between age groups with a one-way ANOVA with factor age group. Significant comparisons (p<.05) were followed up with post-hoc Scheffe’s tests of group differences.

For each age group topographic maps of average absolute spectral power density (μV^2^) were calculated for standard frequency bands for all channels, averaged across the NREM periods of the first 3 sleep cycles. To account for inter-individual variability, normalized maps were also generated by computing the z-score across all good channels for each subject. The correlation of age with power density was calculated for each electrode and Pearson’s correlation coefficient was displayed on topographic maps. Absolute power was log transformed before the correlation with age was computed, to produce normally distributed residuals. To control for multiple comparisons, significant correlations were determined by statistical non-parametric mapping (SnPM) using a single threshold test [44]. To visualize the relationship, the linear correlation was plotted at Fz in each frequency band. To increase the signal-to-noise ratio, statistical analyses of topographies were restricted to channels overlaying the scalp (specifically, 173 channels falling within a plotting radius of 0.57 specified in the topoplot function of the EEGLAB plug-in for MATLAB), with channels on the neck or the face excluded. All statistical procedures were performed using MATLAB (The MathWorks Inc., Natick, MA). To confirm regional differences of the age effect, a mixed ANOVA was conducted with SPSS 22, with age as the between-subjects factor and region as the within subjects factor (2 levels: frontal and left central). Frontal slow wave power was defined as the mean power in a cluster of electrodes encircling Fz. Frontal sigma power, left central slow wave and left central sigma power were defined as the mean power in the electrodes that showed a significant correlation of age with normalized power.

## Results

### Participant Characteristics and Sleep Architecture

92 adults (59 women) met the selection criteria and were included in all analyses. The proportion of women was greater with increasing age (S1 Fig). Polysomnographic characteristics (averaged across the first 3 sleep cycles only) are summarized in Table 1. Increasing age was associated with less deep sleep, evidenced by higher percentages of wake after sleep onset and stages N1 and N2, and lower percentage of stage N3. There was no relationship between age and total sleep time or percent REM sleep during the first 3 sleep cycles. There was no significant interaction effect of sex on these relationships.

**Table 1:** Correlation Between Age and Sleep Architecture.

	mean (SD)	r	p
TST (min)	271.7 (55.8)	-.18	n.s.
WASO (%)	7.9 (6.9)	.48	<.0001
N1 (%)	6.9 (4.2)	.40	<.001
N2 (%)	68.2 (11.5)	.41	<.001
N3 (%)	15.8 (11.1)	-.38	<.0001
REM (%)	19.7 (6.4)	-.16	n.s.

N = 92. r, Pearson product-moment correlation coefficient (2-tailed); p, significance; n.s., non-significant. TST, Total Sleep Time; WASO, Wake After Sleep Onset (% of Time in Bed), N1/2/3/REM(%), NREM stage 1/2/3/REM (% of TST). All variables calculated for the analysis period (first 3 sleep cycles).

### Spectral high density EEG Analysis

**Fig 1:**
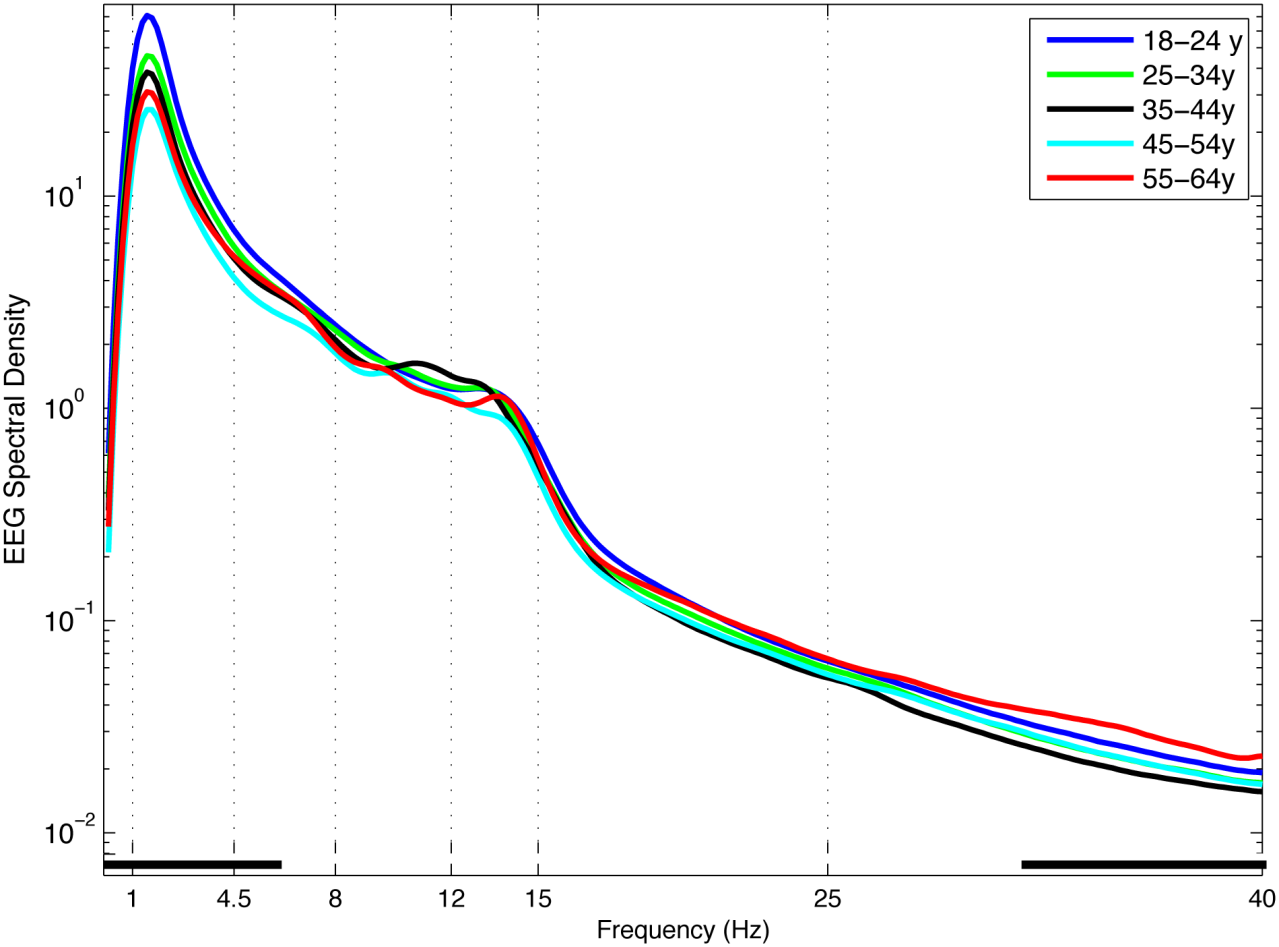
NREM EEG Power Spectra. EEG power spectra (log μV^2^/Hz) for NREM sleep during the first 3 sleep cycles, averaged across 173 scalp electrodes in 1/6 Hz frequency bins. Age groups plotted by decade (aged 18–25 years, dark blue; 25–35 years, green; 35–45 years, black; 45–55 years, light blue; 55–65 years, red). Classically defined frequency bands indicated by vertical dotted lines. Black squares along the x-axis indicate frequency bins in which ANOVA showed a significant effect of age group.

Power spectra, averaged across all channels, are plotted in Fig 1. All age groups showed the classical pattern of NREM spectral activity, with greatest power in the slow wave frequency band (1–4.5 Hz) and a second peak in the sigma band (12–15 Hz). In general, older age groups showed significantly lower power than younger age groups in the slow wave and low theta frequency ranges, and greater power than younger age groups in the high gamma range. No differences between age groups were observed in the high theta, alpha, sigma, or beta frequency ranges.

### High density EEG topography

The topography of absolute EEG spectral power density in standard frequency bands for each age group is shown in S3 Fig. Power in the slow wave range was greatest frontally at all ages. Both frontal and posterior maxima were evident in the theta, alpha and sigma ranges. Representative scatterplots of the correlation between age and EEG power at Fz are shown in S4 Fig.

### Correlation of EEG power with age

**Fig 2:**
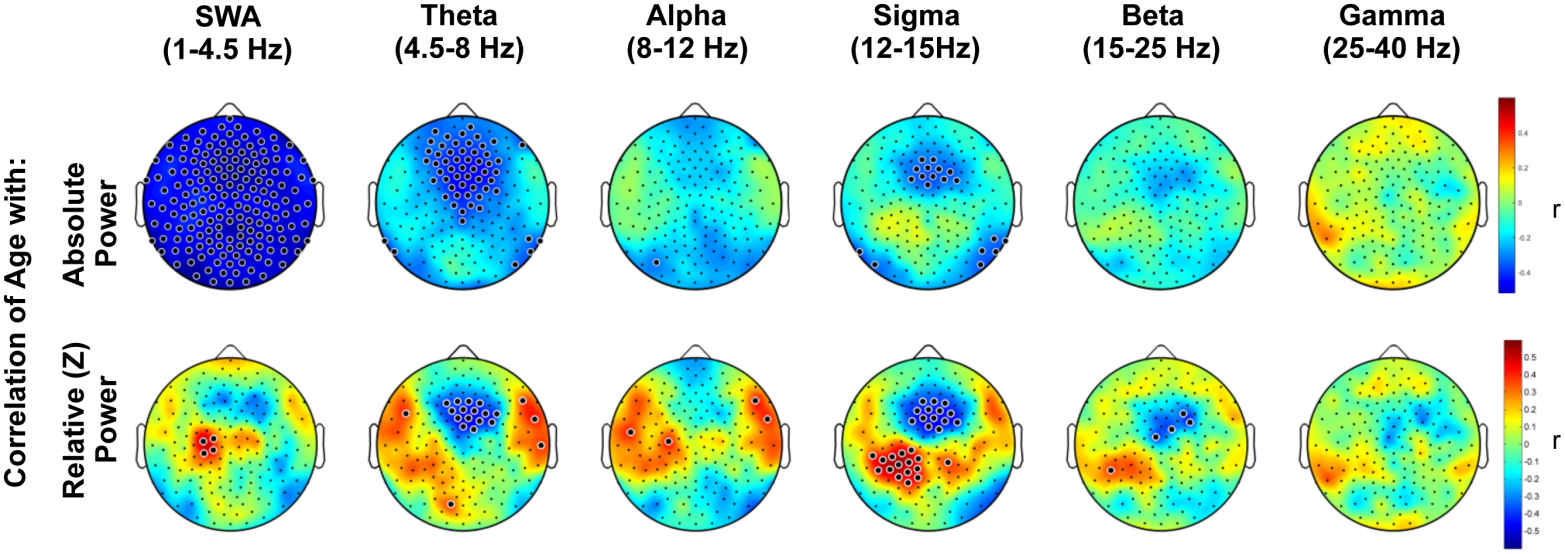
Topography of the correlation of age with NREM EEG power. Topography of the correlation between age and NREM spectral density, averaged across the first three sleep cycles in standard frequency bands. Color represents the coefficient of the correlation between age and power at each electrode. In the upper panel, blue indicates a negative correlation, i.e. a decline in absolute NREM EEG power (log μV^2^) with increasing age. The lower panel plots the coefficient of correlation (r) between age and power (μV^2^) normalized to the scalp mean within an individual. Colors indicate that as age increases, EEG power is increasingly higher (red) or lower (blue) than the scalp mean. Large black dots indicate channels at which the correlation of age and EEG power was significant, accounting for multiple comparisons with statistical nonparametric mapping.

To quantify topographic changes with age, maps were generated for the coefficient of the correlation between power and age at each electrode (Fig 2). When power was expressed as absolute values, slow wave power declined significantly with increasing age across the entire scalp, whereas power in the theta and sigma range declined significantly in a frontal cluster of electrodes adjacent to Fz. There was no significant effect of age on absolute power in the alpha, beta or gamma frequency bands.

**Fig 3:**
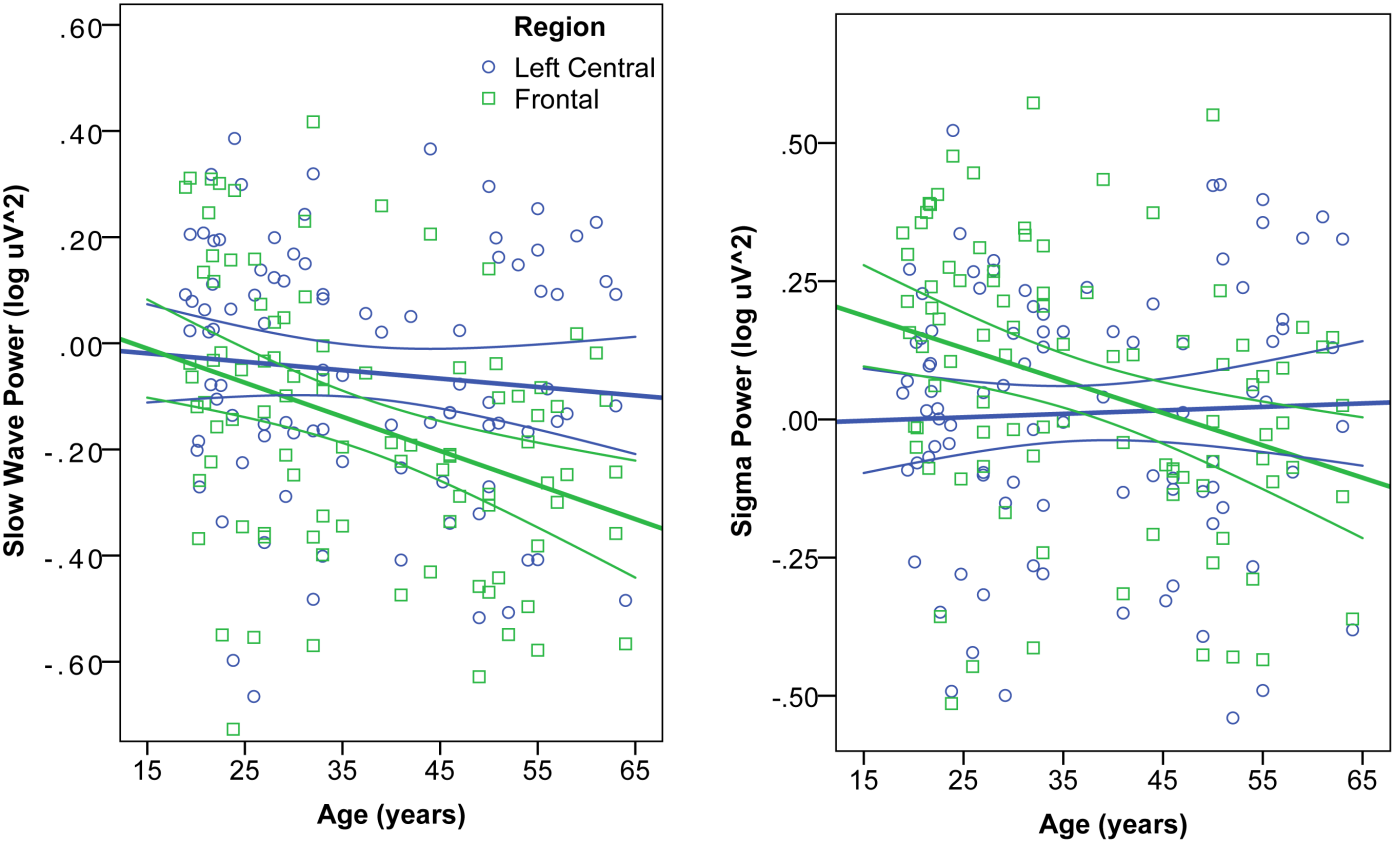
Interaction of age and region on EEG Power. Correlation of age and absolute NREM EEG power (log μV^2^) in the Slow Wave (left) and Sigma (right) frequency bands. In each band, power was averaged in a frontal (blue circles) and left central (green squares) region, defined by clusters of electrodes showing significant correlation of age and normalized power. Mixed ANOVA revealed a significant region x age interaction, such that the age-related decline of EEG power was greater in the frontal than left central region for slow wave and sigma bands.

When EEG power was normalized across the scalp, a left centro-parietal region showed significantly less age-related decline in power than the rest of the scalp. This partial preservation of left centro-parietal power was particularly prominent in the slow wave and spindle bands. The interaction of region and age was confirmed by mixed ANOVA, such that the decline of EEG power with age was greater in the frontal than left central region for the slow wave band [F(2,90) = 28.9, p<.0001] and sigma band [F(2,90) = 30.8, p<.0001] (Fig 3). In general, age-related power declines were greatest frontally, and this effect was statistically significant in the theta and sigma bands. The parietal shift in dominance was driven by a loss of frontal power and preservation of parietal power. It was not driven by an increase in parietal activity, because absolute power did not increase with age at any electrodes.

**Fig 4:**
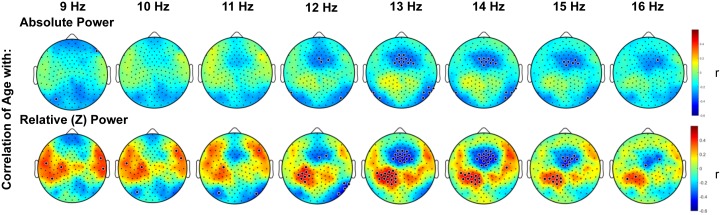
Topography of the correlation of age with NREM EEG spectral density in spindle frequencies. Topography of the correlation between age and NREM EEG spectral density (log μV^2^), in 1 Hz bins of spindle frequencies, averaged across the first three sleep cycles. Color represents the coefficient of the correlation between age and power at each electrode. Small black dots indicate electrode locations. Large black dots indicate electrodes at which the correlation of age and EEG power was significant, accounting for multiple comparisons with statistical nonparametric mapping.

When spindle frequencies were examined in 1 Hz bins (Fig 4), absolute power in faster spindle frequencies (13–16 Hz) declined significantly with increasing age in a frontal cluster adjacent to Fz whereas absolute power of slower spindle frequencies (9–12) showed no significant change with age. Partial preservation of relative power in a centro-parietal region was evident across the spindle band, but was particularly prominent in the 12–15 Hz range.

In all frequency bands the effect of age on EEG topography was similar across sleep cycles, with larger clusters of electrodes showing statistical significance in earlier sleep cycles. Therefore the findings are robust, not driven by one sleep cycle (data not shown). We found no significant effect of sex on the relationship between EEG topography and age (S1 File).

## Discussion

This study examined age-related changes in the topography of sleep EEG power across an age range of 18–65 years. The study design had three key strengths. First, the high spatial resolution of hdEEG used in this study allowed identification of regional effects. Second, age was treated as a continuous variable, rather than comparing groups from opposite ends of the age spectrum. Finally, this study included middle-aged adults, a critical period for brain health that has been largely neglected in studies of sleep and aging.

The effect of age on EEG power varied considerably by region and across frequency bands. We focus our discussion primarily on the slow wave and spindle frequency range, as these waveforms are known to support cognitive processes that change substantially across the lifespan, such as learning and memory.[2,45]

### Scalp-Wide Decline in Absolute Slow Wave Power

With increasing age, slow wave (SW) power declined significantly across the entire scalp. Prior studies reported a frontal dominance of age effects on slow waves,[22,24] based on recordings from only 3 or 4 electrodes along the antero-posterior midline (Fz, Cz, Pz, Oz). The disparity could be due to the EEG referencing techniques used. We used an average reference because this technique has better performance than other commonly used schemes [26,46]. Prior reports referenced to the mastoids, which can distort the EEG signal by removing more physiologic information closer to the mastoids (i.e. central and parietal regions) than in more distant regions (i.e. frontal) [27]. This could explain why frontal effects were more prominent in the prior reports. The scalp-wide topography of SWA decline we observed could be due to high proportions of women in the older age groups in our sample; an age-related decrement in SW incidence was found to be greater frontally in men, but was equally distributed along the antero-posterior axis in women[21]. We found that the effect of age on EEG spectral power did not differ by sex, consistent with some [28] but not all [21,47] previous reports.

Declining SW power in older age is likely driven by anatomical changes. Aging is associated with widespread atrophy of grey matter [14,16], as well as a regional thinning of grey matter that mediates an age-related reduction in SW amplitude and density [3]. In addition, individual differences in SW power correlate with white matter volume and microstructure[48,49]. Slow waves are primarily maintained and synchronized by cortico-cortical connections[34], and more synchronous firing leads to greater summation of electrical potentials, observable as greater SW amplitude and power[5]. Given that DTI measures of white matter structure begin to decline after age 20 years[13], atrophy or degradation of white matter could diminish synchronization, thereby contributing to SW power reduction scalp-wide.

In addition to changes in gross brain structure, the loss of SW power with age may also be driven by synaptic changes. It has been repeatedly suggested that changes in SW power across childhood could be driven by synaptic proliferation and subsequent pruning, motivated by the observation that SW power, synaptic density and brain metabolism follow parallel inverted-U shaped curves up to late adolescence [34,49–51]. It follows that the further decline in SW power across adulthood could be due to continued synaptic loss. However, a recent study testing this hypothesis in mice failed to find an association between SW power and synaptic density across adolescence[52]. Instead, the loss of SW power might result from a more subtle reorganization of cortical circuits. For example, some classes of synapses are more vulnerable to aging than others; thin spines accounted for the majority of spine loss in prefrontal cortex whereas mushroom and stubby spines remained relatively stable with aging in rhesus monkeys[53]. Given that spine morphology affects the strength and dynamics of synaptic transmission, spine changes across aging could result in altered circuit synchronization and lowered SW power.

Slow waves are thought to reflect and participate in plastic processes during sleep[54]. It has already been shown that in a frontal region, age-related declines in SW power correlate with decrements in memory performance[2]. Our findings of a scalp-wide decline in SW power suggest that aging may be associated with impairments in slow-wave mediated plasticity across the entire cortex.

### Frontal Declines in Absolute Theta and Sigma Power

In contrast to the scalp-wide decline in SW power, the age-related decline in theta and sigma power was focused on a frontal region, with no significant declines posteriorly. This pattern is consistent with a previous report using only three mid-line derivations[22]. Grey matter volume correlates with individual differences in maximal theta and sigma power[48]; therefore the frontal pattern of theta and sigma decline observed here could be due to atrophy of frontal grey matter known to accompany aging[14,16]. Oscillations in the theta range (4–7 Hz) during wakefulness promote synaptic plasticity and are linked to memory encoding[55–57], and there is evidence that frontal theta plays a similar role during REM and NREM sleep[58,59]. Thus disruptions of theta oscillations during sleep may contribute to age-related declines in memory performance. The sigma band corresponds to the frequency of spindles, which are discussed in further detail below.

### Preservation of EEG Power in a Left Centro-Parietal Partial Region

Age-related declines in EEG power were less pronounced in a left centro-parietal region. This asymmetry has not been previously described, because previous studies of topography and aging restricted analyses to midline derivations or to one hemisphere. The relative preservation was in the dominant hemisphere (all participants were right-handed). With increasing age, loss of dexterity is more pronounced in the non-dominant hand[60], and atrophy of grey matter is more pronounced in the sensorimotor hand area of the non-dominant hemisphere compared to the dominant hemisphere[61]. Therefore the preservation of EEG power over left centroparietal cortex could be due to a larger neural population contributing to oscillations in that region. Alternatively, the preservation of power may reflect sleep-mediated plastic processes. Regional SW power reflects prior use, for example sleep SW power is increased in parietal cortex following a motor training task[18,19]. Older adults experiencing reduced dexterity in the non-dominant hand may compensate by using the dominant hand more frequently and by learning new motor patterns adapted to their abilities. This motor learning could be reflected in increased parietal SW power.

### Age-Related Decline of Fast Frontal Spindle Band Power, But Not Centroparietal Spindle Band Power

Spindles fall into two distinct classes: fast and slow. Although their precise origins and functions are uncertain, the two classes have different topographies [23,40,43], are differently affected by pharmacological agents [62] and clinical disorders [33,63,64] and make distinct contributions to cognition [65,66]. The frequency cutoffs used to define each class vary throughout the literature; therefore we examined activity in 1Hz bins. We observed the predicted slow-frontal, fast-central topographic distinction in all age groups. Our finding of an age-related decrease of frontal power in faster (13–15 Hz) but not slower (9–12 Hz) spindle frequencies confirms that slow and fast spindles are distinct phenomena that are differently affected by age.

The age-related decline of power in fast spindle frequencies was confined to a frontal region, where fast spindles are usually less prominent [40]. Others have reported age-related declines in spindle band power but the few reports of topographic changes have been mixed. Landolt and Borbely[22], found a posterior shift in slow (10.25-12Hz) but not fast spindle band power in old compared to young adults. Using spindle detection algorithms at 5 midline derivations, Martin et al. [23] found that older adults showed lower spindle density (spindles/min) and amplitude in frontal derivations, whereas an age-related reduction in duration was strongest in posterior derivations. They did not categorize spindles as fast and slow, but did report that spindle frequency was lower in younger adults in posterior derivations during later sleep cycles only. Based on these reports, the frontal loss of fast spindle band power that we observed could be due to reduced spindle amplitude and density, and is unlikely to be due to changes in spindle frequency. Fast spindles are more strongly tied to slow wave upstates than are slow spindles[40]; their loss may be via a shared mechanism with the decline in frontal slow waves.

The precise function of spindles is uncertain but there is evidence for a role in learning and memory and as an attentional ‘gate’ protecting sleep by regulating the salience of external stimuli [67,68]. The functional significance of each spindle class and the implications of selective loss of frontal fast spindle band power are unclear. When a prefrontal region was examined a priori, fewer prefrontal fast spindles (13.5–15 Hz) statistically mediated poorer memory function in older (71.9 ± 6.7 years) compared to younger (20.5 ± 2.1 years) adults [45]. Spindles are generated in the reticular formation of the thalamus and conveyed to the cortex by thalamo-cortical projections [67]. Thus age-related alterations in spindle band power could arise from changes in the thalamus, the cortex or thalamo-cortical projections. Anterior and medial thalamic aspects of the thalamus and their projections to frontal cortex show signs of atrophy with increasing age, while the volume of thalamic projections to parietal, temporal and occipital cortex show no significant relationship with age [16,69]. Age-related deterioration of anterior thalamic nuclei and their projections could therefore impair transmission of fast spindles from the thalamic nuclei to frontal cortex. However the source generators and transmitting fibers of distinct spindle classes are still uncertain and it is unclear why the slow spindle band, which is frontally dominant, was not affected by age.

### Limitations and Strengths of This Study

Limitations of this study include the cross-sectional design and the lack of ad-libitum sleep, which precluded analyses of sleep architecture and time-of-night effects. Data was only analyzed from one night for each participant, however the scalp topography of sleep EEG shows a consistent pattern across nights within individuals [8,9]. Hormonal and menopausal status of women was not assessed in our sample, which could have influenced findings; for example, activity in alpha, sigma and beta frequencies varies across the menstrual cycle [70] and menopause is associated with elevated beta activity [71]. We found, however, that the effect of age on EEG spectral power did not differ by sex (S1 File). This is consistent with previous reports that although EEG spectral density differs between men and women, the effect of age does not vary by sex [28,47].

A key strength of the current study was that it examined brain change across aging by treating age as a continuous variable, whereas previous studies of EEG topography have compared age groups at opposite ends of the age spectrum. Aging is a progressive, rather than step-wise process therefore the treatment of age as a continuous variable is a more appropriate method of evaluating the brain changes associated with aging. It will be important to confirm the age-related changes observed in this cross-sectional dataset with longitudinal studies within individuals.

### Conclusion

This study provides normative data on the effect of age on sleep EEG topography with high spatial resolution. These results are relevant for interpreting sleep EEG changes in disorders that present later in life. For example, the aging-related pattern of scalp-wide SW decline and frontal theta and sigma decline differs markedly from the posterior and parietal EEG changes we have observed in disorders for which older age is a risk factor including OSA [72] and insomnia [64]. Given the substantial effect of age on sleep EEG power and topography, it is important for future studies of sleep EEG to control for age in their statistical models and study design. Furthermore, given the asymmetric effect of age on EEG topography, future study designs should consider regional variation in their measures. The functional and anatomical correlates of age-related EEG topography changes in each frequency band also require further investigation. Understanding normal sleeping brain activity across adulthood provides an important basis from which to explore pathological patterns of brain aging.

## Supporting Information

S1 Fig Distribution of participant age, separated by gender. Males are represented by red dots; women are represented by black dots.
(TIFF)

S2 Fig Distribution of participant age, separated by protocol from which they were sourced. (TIFF)

S3 Fig Topography of absolute EEG spectral density in standard frequency bands, averaged across the first 3 sleep cycles, binned into arbitrary age groups by decade. (TIF)

S4 Fig Correlation of age (years) with NREM EEG spectral density at Fz (log μV^2^), in standard frequency bands, averaged across the first 3 sleep cycles. (TIF)

S1 File Interaction of sex with the effect of age on EEG topography. (DOCX)
