# Supplementary figures and images for: High Resolution Topography of Age-Related Changes in Non-Rapid Eye Movement Sleep Electroencephalography
Source: PLoS One. 2016 Feb 22;11(2):e0149770. doi: 10.1371/journal.pone.0149770 (PMC4764685; doi:10.1371/journal.pone.0149770)

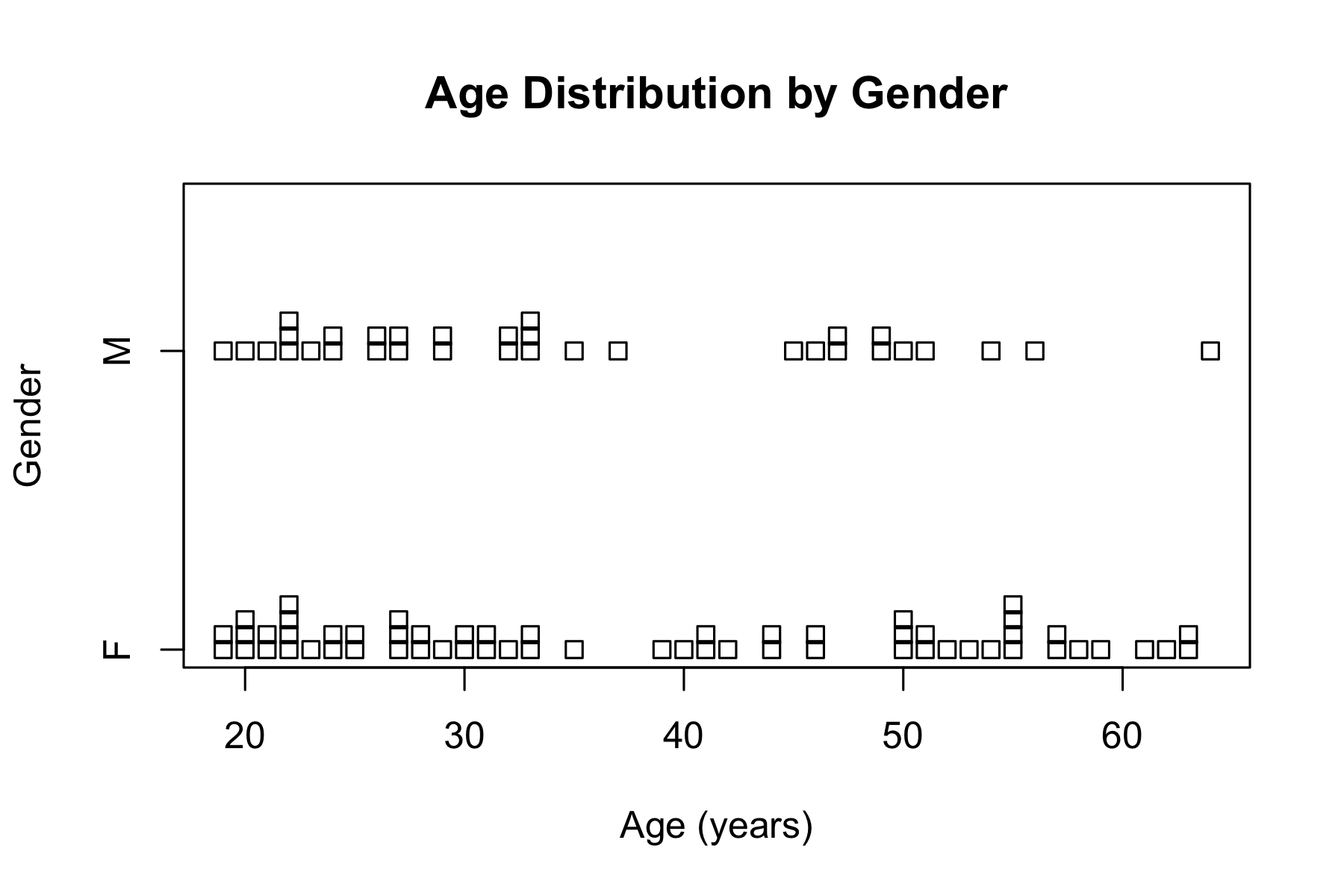

Supplement: S1 Fig — Males are represented by red dots; women are represented by black dots. (TIFF) [file pone.0149770.s001.tiff]

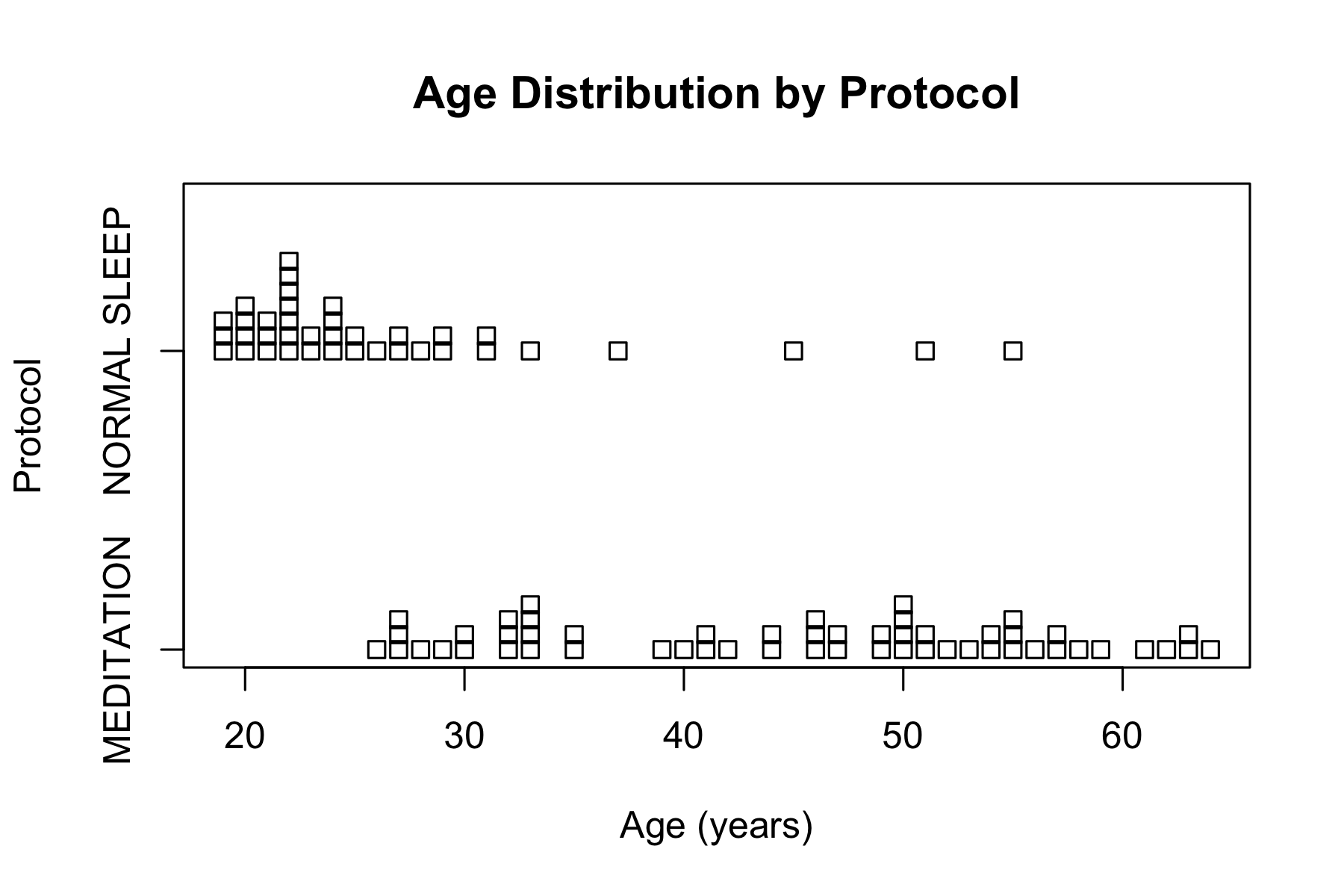

Supplement: S2 Fig — (TIFF) [file pone.0149770.s002.tiff]

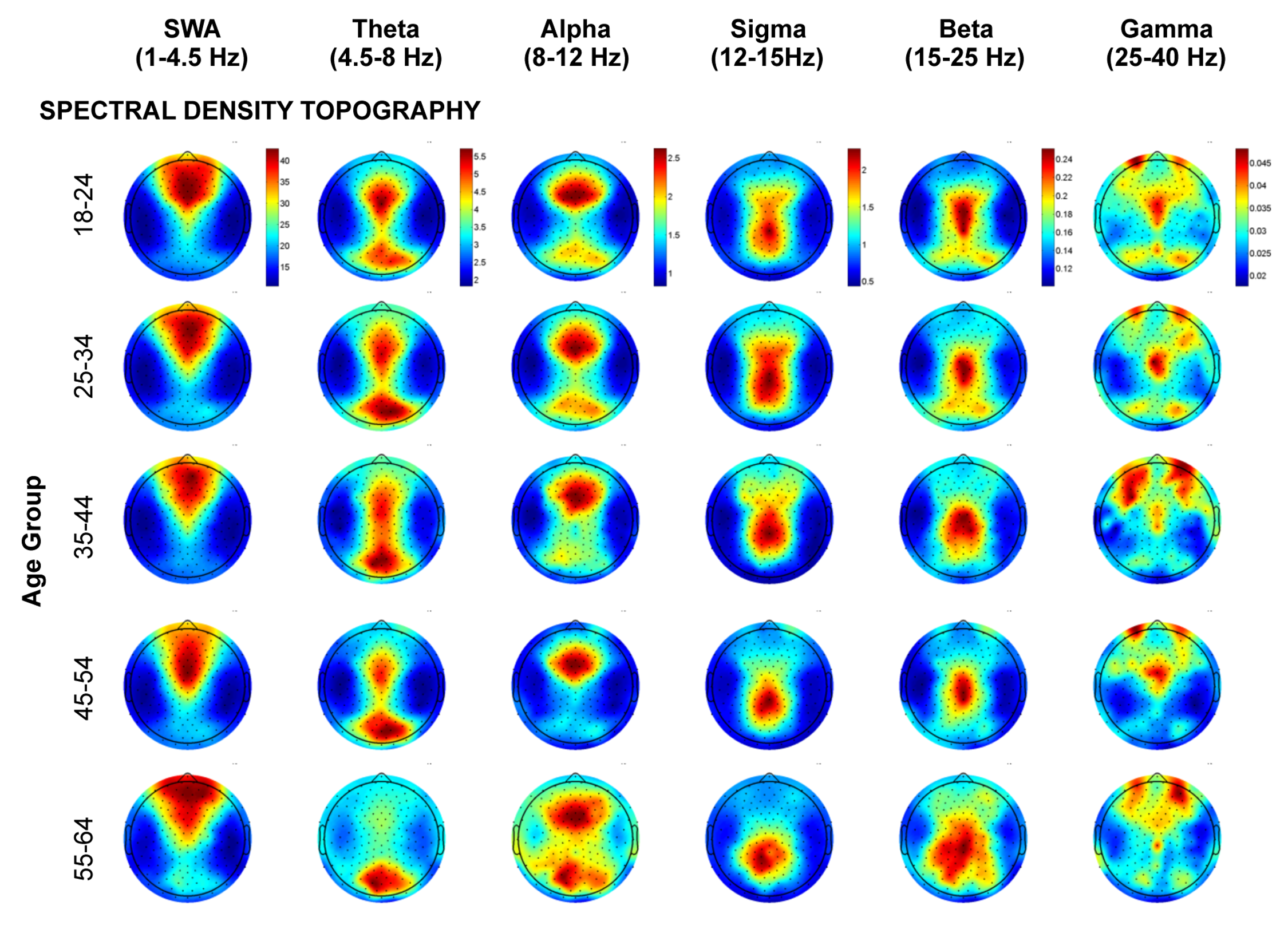

Supplement: S3 Fig — (TIF) [file pone.0149770.s003.tif]

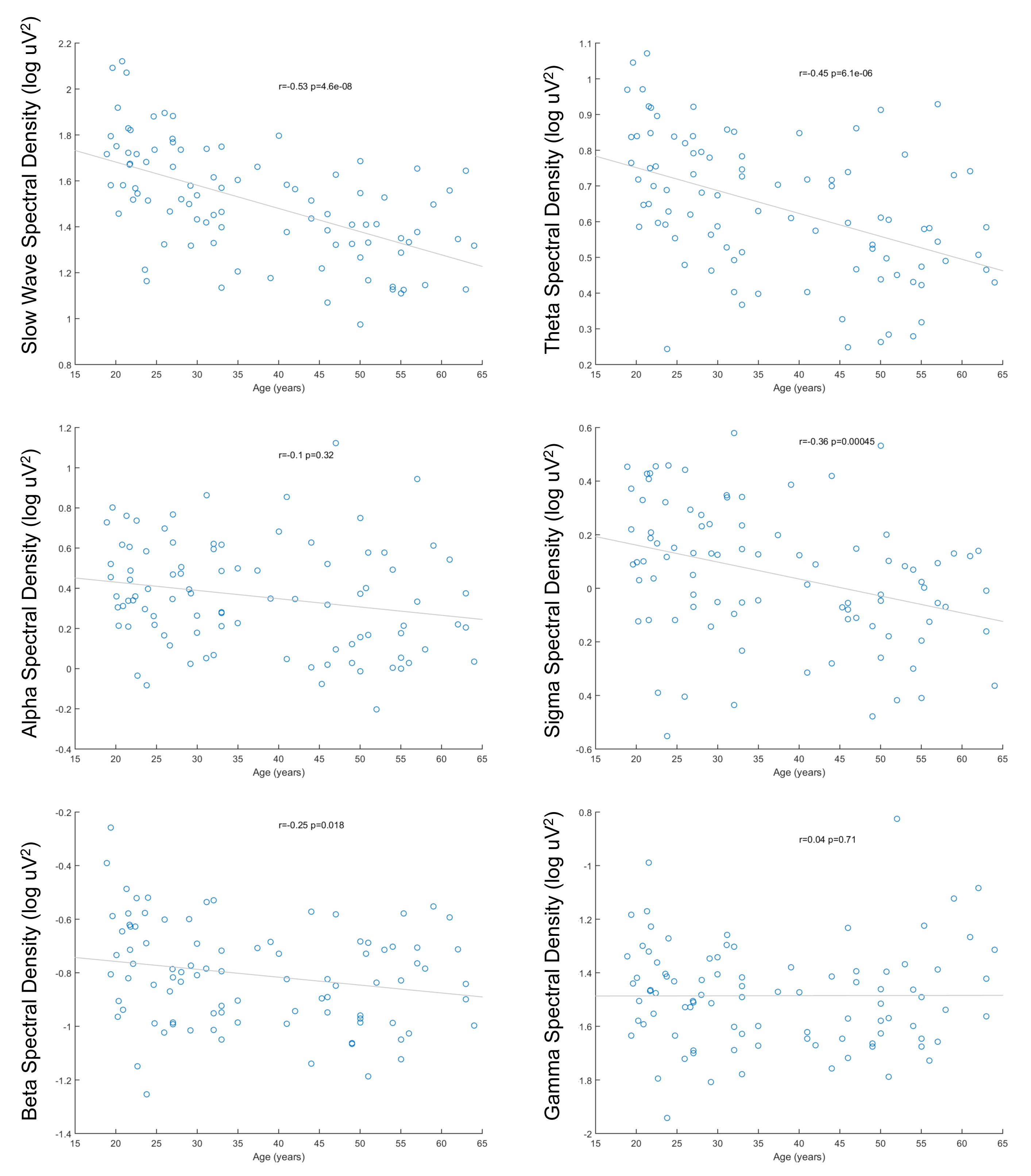

Supplement: S4 Fig — (TIF) [file pone.0149770.s004.tif]
