# Supplementary material for: High Resolution Topography of Age-Related Changes in Non-Rapid Eye Movement Sleep Electroencephalography
Source: PLoS One. 2016 Feb 22;11(2):e0149770. doi: 10.1371/journal.pone.0149770 (PMC4764685; doi:10.1371/journal.pone.0149770)
Supplement: S1 File — (DOCX) [file pone.0149770.s005.docx]

**Interaction of sex with the effect of age on EEG topography.**

Given the lack of men aged over 50 years (n=5), these analyses should be considered exploratory only.

To investigate the interaction of sex with the effect of age on EEG topography, we performed the topographic analysis separately in women and men, and observed a similar topography of the correlation of age and EEG power. The small sample of men (n=33), especially in the older age range (see supplemental figure 1) may account for the lack of statistical significance in men.

To reduce the number of statistical tests, we restricted our analyses to regional difference scores in the slow wave and sigma bands. As described in the body of the manuscript, we found that the effect of age on slow wave and sigma power was greater frontally than centrally, such that the decline of EEG power with age was greater in the frontal than left central region (Figure 3 of the main manuscript). After averaging EEG power in frontal and central clusters (defined under Methods), we subtracted frontal power from central power to create a difference score for each individual, in each band. We then regressed the difference score on age, sex and age*sex.

In the slow wave band there was a significant effect of age, no significant effect of sex, and a marginally significant interaction effect of sex and age on the difference between frontal and central power (b=.01, F(3,88)=8.1, p=.047) (Figure 2). Testing the simple effects revealed that the difference between frontal and central power increased significantly with age in females (b=.013, F(1,57)=16.7, p<.001) but not males (b=.003, F(1,31)=.93, p=.34). Sex did not significantly interact with the relationship between age and sigma topography (b=.012, F(3,88)=10.1, p=.18) (Figure 3).


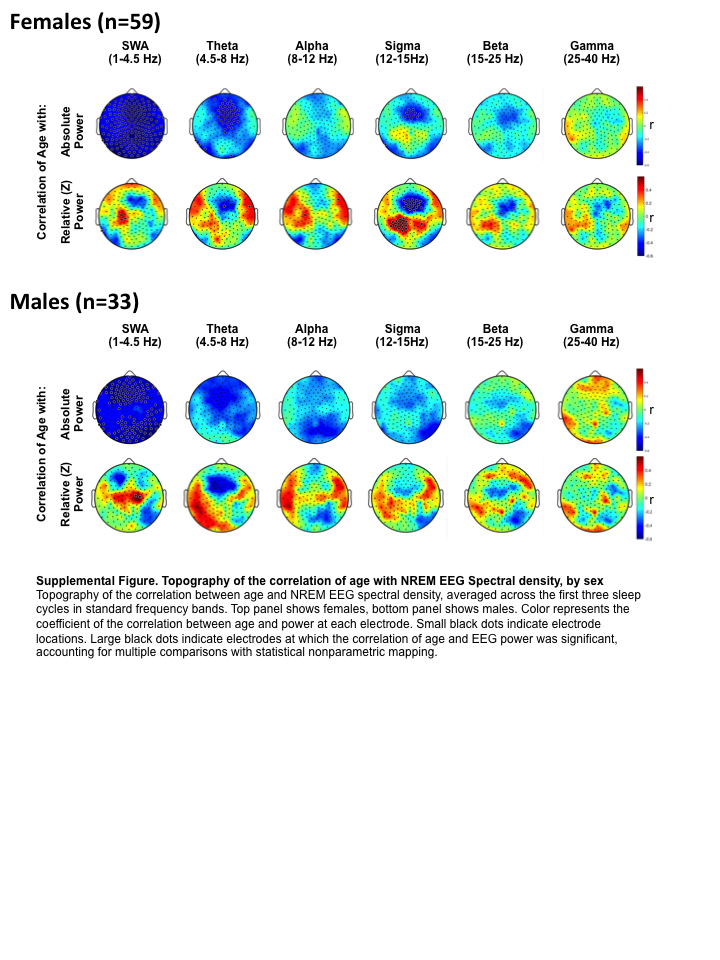


**Figure 1. Topography of the correlation of age with NREM EEG Spectral density, by sex.** Topography of the correlation between age and NREM EEG spectral density, averaged across the first three sleep cycles in standard frequency bands. Top panel shows females, bottom panel shows males. Color represents the coefficient of the correlation between age and power at each electrode. Small black dots indicate electrode locations. Large black dots indicate electrodes at which the correlation of age and EEG power was significant, accounting for multiple comparisons with statistical nonparametric mapping.


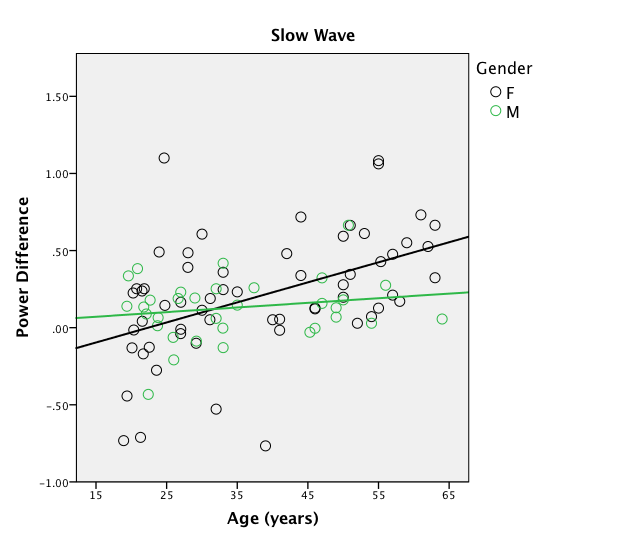


**Figure 2.** Effect of age and sex on the difference between frontal and central SW power.


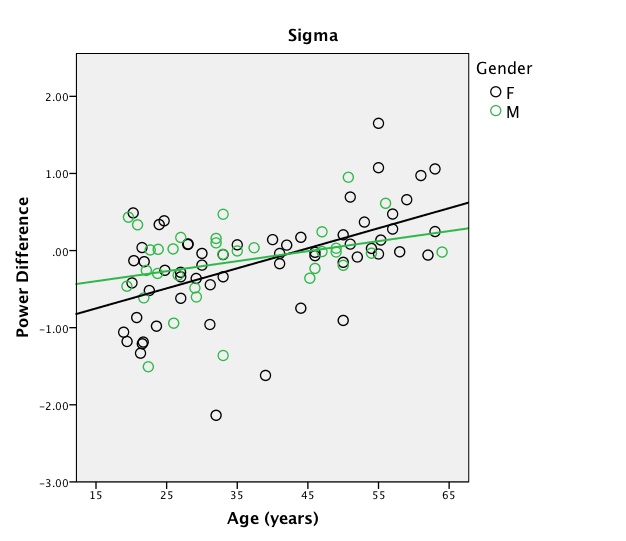


**Figure 3.** Effect of age and sex on the difference between frontal and central sigma power.
